# Prediction of Maternal Hemorrhage Using Machine Learning: Retrospective Cohort Study

**DOI:** 10.2196/34108

**Published:** 2022-07-18

**Authors:** Jill M Westcott, Francine Hughes, Wenke Liu, Mark Grivainis, Iffath Hoskins, David Fenyo

**Affiliations:** 1 Division of Maternal-Fetal Medicine, Department of Obstetrics and Gynecology New York University Langone Health New York, NY United States; 2 Montefiore Medical Center Albert Einstein College of Medicine Bronx, NY United States; 3 Institute for Systems Genetics New York University Grossman School of Medicine New York, NY United States; 4 Department of Biochemistry and Molecular Pharmacology New York University Grossman School of Medicine New York, NY United States

**Keywords:** predictive modeling, maternal morbidity, postpartum hemorrhage, machine learning, obstetrics, pregnancy, post partum, maternal

## Abstract

**Background:**

Postpartum hemorrhage remains one of the largest causes of maternal morbidity and mortality in the United States.

**Objective:**

The aim of this paper is to use machine learning techniques to identify patients at risk for postpartum hemorrhage at obstetric delivery.

**Methods:**

Women aged 18 to 55 years delivering at a major academic center from July 2013 to October 2018 were included for analysis (N=30,867). A total of 497 variables were collected from the electronic medical record including the following: demographic information; obstetric, medical, surgical, and family history; vital signs; laboratory results; labor medication exposures; and delivery outcomes. Postpartum hemorrhage was defined as a blood loss of ≥1000 mL at the time of delivery, regardless of delivery method, with 2179 (7.1%) positive cases observed. Supervised learning with regression-, tree-, and kernel-based machine learning methods was used to create classification models based upon training (21,606/30,867, 70%) and validation (4630/30,867, 15%) cohorts. Models were tuned using feature selection algorithms and domain knowledge. An independent test cohort (4631/30,867, 15%) determined final performance by assessing for accuracy, area under the receiver operating curve (AUROC), and sensitivity for proper classification of postpartum hemorrhage. Separate models were created using all collected data versus models limited to data available prior to the second stage of labor or at the time of decision to proceed with cesarean delivery. Additional models examined patients by mode of delivery.

**Results:**

Gradient boosted decision trees achieved the best discrimination in the overall model. The model including all data mildly outperformed the second stage model (AUROC 0.979, 95% CI 0.971-0.986 vs AUROC 0.955, 95% CI 0.939-0.970). Optimal model accuracy was 98.1% with a sensitivity of 0.763 for positive prediction of postpartum hemorrhage. The second stage model achieved an accuracy of 98.0% with a sensitivity of 0.737. Other selected algorithms returned models that performed with decreased discrimination. Models stratified by mode of delivery achieved good to excellent discrimination but lacked the sensitivity necessary for clinical applicability.

**Conclusions:**

Machine learning methods can be used to identify women at risk for postpartum hemorrhage who may benefit from individualized preventative measures. Models limited to data available prior to delivery perform nearly as well as those with more complete data sets, supporting their potential utility in the clinical setting. Further work is necessary to create successful models based upon mode of delivery and to validate the findings of this study. An unbiased approach to hemorrhage risk prediction may be superior to human risk assessment and represents an area for future research.

## Introduction

Postpartum hemorrhage is the leading cause of maternal mortality worldwide [1]. In the United States, the rate of postpartum hemorrhage continues to rise, complicating nearly 3% of deliveries [2]. Mothers with severe hemorrhage may require blood transfusion, hysterectomy, or intensive care unit admission with a select number of cases proving fatal. Postpartum hemorrhage that leads to blood transfusion is the leading cause of severe maternal morbidity in the United States [3]. Stewardship of blood resources and minimizing hemorrhage-related morbidity remain ongoing efforts as blood transfusion is not without risk. By predicting patients at risk for significant blood loss, prophylactic measures may be instituted to avoid maternal morbidity and mortality.

A number of risk factors for postpartum hemorrhage have been established, including previous postpartum hemorrhage, multifetal gestation, pre-eclampsia, augmented labor, fetal macrosomia, operative vaginal delivery, and complex lacerations, as well as other factors [4]. Previous models for prediction of postpartum hemorrhage have been developed [5-7], but validation of these among different populations and at different time points within the labor process has been limited. A machine learning study using administrative data provided poor discrimination for predicting the need for hospital readmission due to postpartum hemorrhage in the first 12 weeks postpartum [8]. Prediction of postpartum hemorrhage remains a challenge for the obstetric provider, and further work is necessary using modern modeling methods.

The field of machine learning has recently seen a rapid development of methods that support unbiased learning from data. Supervised learning involves processing information to predict from examples with a known outcome, often for the purpose of estimating risk in examples where the outcome is not known [9]. Multiple applications for machine learning exist within medicine; however, to date, they have not been widely used in the field of obstetrics. By using the power of modern predictive modeling for postpartum hemorrhage, we aim to better identify those patients at increased risk for obstetric hemorrhage to avoid maternal morbidity and mortality. Identifying patients at the highest risk of postpartum hemorrhage will enable providers to reduce the cost and morbidity associated with postpartum hemorrhage and ultimately improve patient outcomes.

## Methods

### Ethics Approval

Institutional Review Board approval was obtained from New York University Langone Health (approval number s18-01798).

### Study Population

This was a retrospective cohort study conducted at a single tertiary care center. Women aged 18 to 55 years delivering at New York University Langone Health Tisch Hospital from July 1, 2013, to October 31, 2018, were included for analysis. Patients not meeting age parameters as well as those cases in which a blood loss value was either not available or not recorded were excluded. All patients not meeting exclusion criteria were included in the study.

### Study Design and Model Development

A total of 497 variables were collected from unique sources within the electronic medical record including the following: demographic information; obstetric, medical, surgical, and family history; vital signs; laboratory results; labor exposures; and delivery outcomes (Multimedia Appendix 1). Postpartum hemorrhage was defined as a blood loss of ≥1000 mL at the time of delivery, as recommended by the American College of Obstetricians and Gynecologists revitalize program [10].

The delivery cohort was randomly split into training (21,606/30,867, 70% of total cohort) and validation (4630/30,867, 15%) sets for model creation. Using the R software (version 3.5.1; R Foundation for Statistical Computing), supervised learning with regression-, tree-, and kernel-based machine learning methods was used to create classification models, using each method for every model assessed. The models were tuned using recursive feature selection, selection by filtering, observing feature importance, and domain knowledge. The model parameters were customized and examined to produce optimal results. An independent test cohort (4631/30,867, 15%) determined the final performance by assessing for accuracy, area under the receiver operating curve (AUROC), and sensitivity for the proper classification of postpartum hemorrhage.

The initial model included variables that contained information that would be feasible to obtain prior to delivery (ie, relevant historical information, objective data present within the inpatient and outpatient chart, and diagnoses associated with the patient’s delivery encounter entered within 24 hours following delivery). A secondary model was created limited to data strictly expected to be available prior to the second stage of labor or at the time of decision to proceed with cesarean delivery, as this was likely the more clinically useful tool. Additional models were created for patients undergoing cesarean and vaginal delivery.

The selection of appropriate variables for inclusion was made by a single obstetric provider with experience and knowledge of the electronic medical record. The number of initial variables in each model differed according to clinical applicability. The variables were processed according to the provider’s assessment of the clinical scenario noted for each patient.

## Results

A total of 30,867 patients met the inclusion criteria, and 2179 (7.1%) cases met the criteria for postpartum hemorrhage. Patient characteristics are detailed in Table 1. Cesarean delivery was noted in 27.6% (n=8534) of the patients, and unknown mode of delivery in 0.1% (n=19), with the remainder (n=22,314, 72.3%) undergoing vaginal delivery. The rate of postpartum hemorrhage by mode of delivery was 20.8% (1776/8534) for cesarean delivery, 21.1% (4/19) for unknown mode of delivery, and 1.8% (399/22,314) for vaginal delivery. The average gestational age was 274.6 (range 107-303) days, and the average patient age was 32.7 (range 18-55) years. The delivery cohort was split into training, validation, and test cohorts for initial model creation containing 21,606 (70%), 4630 (15%), and 4631 (15%) patients, respectively.

**Table 1:** Patient cohort characteristics.

Variable	Value	
	Overall (N=30,867)	PPH^a^ (n=2179)
Age (years), mean (range, SD)	32.7 (18-55, 5.3)	34.3 (18-54, 5.4)
Gestation (days)	274.6	272.2
BMI (kg/m^2^)	28.5	30.0
Vaginal delivery, n (%)	22,314 (72.3)	399 (18.3)
Cesarean delivery, n (%)	8534 (27.6)	1776 (81.5)
Unknown mode of delivery, n (%)	19 (0.1)	4 (0.2)
Operative vaginal delivery, n (%)	1502 (4.9)	51 (2.3)
Vaginal birth after cesarean, n (%)	1019 (3.3)	24 (1.1)
Multiple gestation, n (%)	143 (0.5)	35 (1.6)
Current tobacco use, n (%)	142 (0.5)	10 (0.5)
Maternal diabetes, n (%)	1544 (5.0)	172 (7.9)
Maternal pre-eclampsia or eclampsia, n (%)	508 (1.6)	65 (3.0)
IUFD^b^, n (%)	111 (0.4)	11 (0.5)
Labor induced, n (%)	16,962 (55.0)	927 (42.5)
Primiparous, n (%)	16,858 (54.6)	1404 (64.4)
Primary cesarean delivery, n (%)	7070 (82.8)	1564 (88.1)
Cesarean performed prior to labor or rupture, n (%)	4801 (56.3)	880 (49.5)

^a^PPH: postpartum hemorrhage.

^b^IUFD: intrauterine fetal demise.

The initial model included a total of 280 variables. Logistic regression, random forest, gradient boosted decision trees), and support vector machine models were generated to create a representative sample of different methods. Gradient boosted decision trees achieved the best discrimination among the initial models, performing with an AUROC of 0.979 (95% CI 0.971-0.986) and an accuracy of 98.1%. Sensitivity for this model was 0.763 (95% CI 0.712-0.809, Table 2). Other models performed less successfully. The optimal model included 212 features (Multimedia Appendix 2).

**Table 2:** Optimal performance of all models using gradient boosted decision trees.

Model	Accuracy	AUROC^a^	Sensitivity
Initial	0.981	0.979	0.763
Second stage	0.980	0.955	0.737
Cesarean delivery	0.818	0.737	0.320
Vaginal delivery (1 hour^b^)	0.982	0.837	0.254
Vaginal delivery (second stage)	0.983	0.846	0.254

^a^AUROC: area under the receiver operating curve.

^b^Within 1 hour of presumed admission for vaginal delivery.

The data set was then trimmed to include only those variables (123 in total) available prior to the second stage of labor or at the time of decision to proceed with cesarean delivery. A similar representative sample of modeling methods was used, with gradient boosted decision trees again achieving the best discrimination, noting an AUROC of 0.955 (95% CI 0.939-0.970) and an accuracy of 98.0%. Sensitivity for this model was 0.737 (95% CI 0.684-0.785; Table 2). This model included a total of 28 features (Textbox 1). The most important features included body mass index, admission hematocrit, cesarean delivery prior to labor or rupture, scheduling status of cesarean delivery, and admission platelet count.

**Features included in the optimal second stage model.**

**Laboratory components**

- Eosinophils (absolute)
- Hematocrit
- Hemoglobin
- Lymphocytes (absolute)
- Lymphocytes (percent)
- Mean corpuscular hemoglobin
- Mean corpuscular hemoglobin concentration
- Mean corpuscular volume
- Mean platelet volume
- Monocytes (percent)
- Neutrophils (absolute)
- Neutrophils (percent)
- Platelet count
- Red cell distribution width (standard deviation)
- Red blood cell count
- White blood cell count

**Nonlaboratory components**

- Patient age
- Gestational age
- Systolic blood pressure
- Diastolic blood pressure
- BMI
- Pulse oximetry
- Temperature
- Live birth count
- Baseline fetal heart rate
- Amniotic fluid color
- Cesarean delivery prior to labor or rupture
- Cesarean delivery scheduling status

Additional models focused on classifying patients by mode of delivery. A model examining those patients ultimately delivered by cesarean delivery (n=8534) was created using information available at the time of decision to proceed with cesarean delivery (ie, within 1 hour after presentation for scheduled procedure or following attempted vaginal delivery prior to proceeding to the operating room). A total of 173 variables were included. Using gradient boosted decision trees, the highest performing model contained 76 variables (Multimedia Appendix 3) and achieved an AUROC of 0.737 (95% CI 0.703-0.772). Accuracy of 81.8% and a lower sensitivity of 0.320 were noted (95% CI 0.266-0.377; Table 2).

Two models using data of those patients who underwent vaginal delivery (n=22,333) were also created. The first focused on information available within 1 hour of admission for presumed vaginal delivery and examined 127 variables. The model achieved excellent discrimination, noting an AUROC of 0.837 (95% CI 0.782-0.893) and accuracy of 98.2%; however, sensitivity remained low at 0.254 (95% CI 0.155-0.375). The optimal model included 7 variables (Multimedia Appendix 4). The second model used information available prior to the second stage of labor; thus, it comprised variables related to the patient’s labor course, including medication exposure and induction method, if applicable. A total of 176 features were included, resulting in an optimal model with an AUROC of 0.846 (95% CI 0.790-0.902) and accuracy of 98.3%. Sensitivity was again 0.254 (95% CI 0.153-0.379), and the final model included 92 features (Table 2; Multimedia Appendix 5). Both of these models were achieved using gradient boosted decision trees. Third trimester and admission hemoglobin or hematocrit were among the most important features in both vaginal delivery models.

## Discussion

### Principal Findings

Our study successfully produced a model for predicting postpartum hemorrhage in patients undergoing obstetric delivery. When using only the data available prior to the second stage of labor or at the time to proceed with cesarean delivery, we achieved nearly equal discrimination and sensitivity compared to our more robust initial model, successfully predicting nearly 3 out of every 4 patients who had a postpartum hemorrhage.

Many previously identified risk factors for postpartum hemorrhage were not included in the final model, including multiple gestation, operative vaginal delivery, and history of postpartum hemorrhage, among others. This indicates that many of these factors may not be as contributory to postpartum hemorrhage risk as previously believed, but further work is necessary.

### Prior Results

Postpartum hemorrhage is a known cause of significant maternal morbidity and mortality in the United States and remains difficult to predict. Few existing studies have used machine learning methods to identify patients at risk for postpartum hemorrhage with minimal success [5-8]. A recently published model used a large cohort from the US Consortium for Safe Labor and achieved excellent discrimination, although its utility in the clinical setting is limited given its retrospective nature without prospective validation [11]. This study used 55 predictor variables, indicating a less robust data set than what was curated for our model. Our study represents the largest cohort to date to generate a predictive risk model using data directly abstracted from the electronic medical record that is applicable in a targeted population.

When stratified by delivery method, our models noted a decreased sensitivity. While this may appear in contradiction to the expected results, it is understandable because the majority of postpartum hemorrhages occurred in those patients who underwent cesarean delivery. This is further reflected by examining the most important features in our final second-stage model.

### Clinical Implications

The ability to predict patients at risk for postpartum hemorrhage using readily available information represents an area of substantial clinical opportunity. Integrating a model such as ours into clinical practice will give providers the real-time capability to assess a patient’s risk of hemorrhage. Targeted intervention, such as prophylactic administration of uterotonic medication, availability of blood products, and even potentially transferring patients to a center offering a higher level of maternal care [12] is a consideration for those deemed at risk.

### Strengths and Limitations

The strengths of this study include the use of modern supervised machine learning techniques in a clinical condition that has not been extensively explored with this approach. This data set represents the largest directly derived cohort to use these techniques. Additionally, the inclusion of nearly 500 variables in the data set provides a robust cohort from which to create the model, and this size has not been previously seen in the literature. As machine learning methods are centered upon improving performance with increasing inputs, this lends to a superior model. Since having a large number of overfitting variables is a concern, this must be considered when determining the optimal model. A slight decrease in accuracy may be necessary to select a model with less concern for overfitting. The use of independent validation and test cohorts also supports the strength and lack of bias in our model.

Limitations include the retrospective nature of this study as well as the use of a population from a single tertiary center. Given regional variations in patient populations, our results may not be generalizable to the US population at large, and we do note a higher rate of postpartum hemorrhage in our cohort than previously described. It is unclear why the rate was higher in our population, but it may be partially explained by the referral nature of our tertiary center, leading to care of a larger number of patients at high risk at baseline. Further validation with an outside cohort and prospective validation among our patient population is necessary.

The use of the electronic medical record is an additional limitation to our study. Differences or duplications in both location and format of inputs have the potential to impair the accuracy of our abstracted data. We are unable to assess the performance or bias of this model across race as this is a variable inputted by the clinical staff; thus, we are unable to validate its accuracy. The variables related to diagnosis codes are entirely dependent upon provider input, and all applicable conditions may not have been entered. However, with a large data set, machine learning algorithms should be able to overcome this deficit as features with a high level of contribution to the outcome should persist when feature selection is implemented.

The class imbalance of positive or negative cases for postpartum hemorrhage in the data set is inevitable given the relatively low incidence of this condition in clinical practice. This was particularly evident in the support vector machine models where every patient was predicted not to be at risk for postpartum hemorrhage. The use of a weighted loss could be considered to compensate for this imbalance.

### Conclusions

In conclusion, machine learning methods are a less used approach in obstetrics and can be used to identify women at risk for postpartum hemorrhage who may benefit from individualized preventative measures. Models limited to data available prior to delivery perform nearly as well as those with more complete data sets, identifying nearly three-quarters of patients at risk, supporting their potential utility in the clinical setting.

## Appendix

Multimedia Appendix 1 Supplementary material 1: 497 variables abstracted.

Multimedia Appendix 2 Supplementary material 2: 212 variables abstracted for initial model (*** = top 5 importance).

Multimedia Appendix 3 Supplementary material 3: 76 variables abstracted for cesarean delivery model (*** = top 5 importance).

Multimedia Appendix 4 Supplementary material 4: 7 variables abstracted for 1 hour vaginal delivery model (*** = top 5 importance).

Multimedia Appendix 5 Supplementary material 5: 92 variables abstracted for 2nd stage vaginal delivery model (*** = top 5 importance).

## Abbreviations

AUROC: area under the receiver operating curve
