# Supplementary material for: Prediction of Maternal Hemorrhage Using Machine Learning: Retrospective Cohort Study
Source: J Med Internet Res. 2022 Jul 18;24(7):e34108. doi: 10.2196/34108 (PMC9345059; doi:10.2196/34108)
Supplement: Multimedia Appendix 1 [file jmir_v24i7e34108_app1.docx]

Supplementary material 1: 497 variables abstracted

| Patient ID | Misoprostol | DIC |
| --- | --- | --- |
| Patient MRN | Nitroglycerin | Endometritis |
| Admission Date | TXA | Excess_weight_gain |
| Delivery Date | NUM_DELIVERIES | Failed_operative_VD |
| GA from Clarity | RESULT_DATE_LABS_PRENATAL | Fibroids |
| GA_WEEKS | GLUCOSE CHALLENGE, PREGNANCY | Fourth_degree |
| GA_DAYS | GLUCOSE TOLERANCE TEST 1 HOUR | GBS |
| GA_IN_DAYS | GLUCOSE TOLERANCE TEST 2 HOUR | GDM |
| 6-MP | GLUCOSE TOLERANCE TEST 3 HOUR | GHTN |
| Allergy | GLUCOSE TOLERANCE TEST FASTING | Grand_multip |
| Amoxicillin | GLUCOSE, TOLERANCE | H_o_PPH |
| Ampicillin | GLUCOSE,TOLERANCE | Hemorrhage |
| Anti-coagulant | GROUP B STREP ANTIGEN | Hypertension |
| Anti-epileptic | HEPATITIS B SURFACE ANTIGEN | IUFD_or_demise |
| Antibiotic | HIV 1 AND 2 ABS-INDEX VALUE | IUGR |
| Antiemetic | HIV 1 AND 2 ABS, QUALITATIVE | Invasive_placenta |
| Antihypertensive | HIV 1 AND 2 AG/AB, 4TH GEN | LGA |
| Antipsychotic | HIV 1 AND 2 ANTIBODY DIFFERENTIATION | Low-lying |
| Antiretroviral | HIV 1 AND 2 ANTIBODY DIFFERENTIATION INTERPRETATION | Macrosomia |
| Antiviral | HIV 1 AND 2 PRESENCE BY RAPID IMMUNOASSAY, POC | Malpresentation |
| Aspirin | HIV 1 ANTIBODY BY MULTISPOT | Oligo |
| Benzodiazepine | HIV 1/2 AB NOTE | Operative_VD |
| Betamethasone | HIV 2 ANTIBODY | Other_multiple |
| Buspirone | HIV 2 ANTIBODY BY MULTISPOT | PPCM |
| Cefazolin | HIV 2 ANTIBODY SUPPLEMENTAL | PPH |
| Clindamycin | HIV 4TH GEN COMMENT | PPROM |
| Colace | HIV AB, WESTERN BLOT | PROM |
| DDAVP | HIV DIAGNOSTIC TESTING ALGORITHM FINAL INTERPRETATION | PTL |
| Dexamethasone | HIV INTERPRETATION | Poly |
| Digoxin | HIV MULTISPOT | Poor_weight_gain |
| Ephedrine | HIV-1 AB,WESTERN BLOT | Pre-eclampsia_or_eclampsia |
| Epidural opioid | HIV-1 AND 2 ANTIBODY | Previa |
| Erythromycin | HIV-1 ANTIBODY | Retained_placenta |
| Factor VIIa | HIV-1 FINAL INTERPRETATION | SGA |
| Flecainide | HIV-1/2 AB EIA SCREEN | Shock |
| Fludrocortisone | HIV1,2 ANTIBODY | Shoulder_dystocia |
| Gentamycin | RUBELLA ANTIBODY, IGG | Succenturiate |
| H2 blocker | RUBELLA IGG QUANTITATION | TRAP |
| Heparin | RUBELLA IGG SCREEN | TTTS |
| Hydralazine | RUBEOLA IGG ANTIBODY | Third_degree |
| Hydroxychloroquine | STREP GROUP B SCREEN, VAGINAL | Thrombocytopenia |
| IBD drugs | STREP GROUP B, CULTURE | Triplet |
| IV opioid | STREP GROUP B, GENITAL CULTURE | Twins |
| Imuran | SYPHILIS IGG-TREPONEMA PALLIDUM | Uterine_rupture_y |
| Indocin | THYROID STIMULATING HORMONE | VWD |
| Inhaled anticholinergic | TSH HIGH SENSITIVITY | Vanishing_Twin_or_IUFD_or_reduction |
| Inhaled steroids | URINE CULTURE SCREEN | Vasa |
| Insulin | VARICELLA ZOSTER IGG | Zika |
| Insulin NPH | HEMATOCRIT_FIRST_TRIMESTER | Admsn Date |
| Insulin aspart | HEMOGLOBIN_FIRST_TRIMESTER | Mother Name |
| Insulin detemir | HEMATOCRIT_SECOND_TRIMESTER | Mother MRN |
| Insulin glargine | HEMOGLOBIN_SECOND_TRIMESTER | Patient Age |
| Insulin lispro | HEMATOCRIT_THIRD_TRIMESTER | Blood Type |
| Insulin regular | HEMOGLOBIN_THIRD_TRIMESTER | EBL |
| Labetalol | DAYS_BETWEEN_ADMISSION_AND_DELIVERY | Blood Loss |
| Lithium | BIRTH_DATE | Bld. Loss |
| Long-acting beta agonist | PAT_NAME | Episiotomy |
| Lovenox | MARITAL_STATUS | Onset Date |
| Magnesium | RACE | Onset Time |
| Mesalamine | ETHNICITY | Induction |
| Methylprednisolone | AGE_AT_DELIVERY | Augmentation |
| Miralax | ALLERGIES | Crv. Rp. Dt. |
| Montelukast | SMOKING_TOB | Crv. Rp. Tm. |
| Nifedipine | ALCOHOL_USE | Crv. Rp. Typ. |
| Oral glycemic | ILL_DRUG_USER | Rupture Date |
| Oral opioid | MEDICAL_HX_FAMILY_HISTORY | Rupture Time |
| Oxytocin | AMA_x | Rupture Type |
| PCN | APLS_or_antibodies | Fluid Color |
| PPI | Abnormal_cervical_findings | 2nd Stg. Dt |
| PTU | All_thal | 2nd Stg. Tm |
| Phenylephrine | Alpha_thal_or_trait | Lacerations |
| Potassium replacement | Asthma | Repair Suture |
| Prednisone | BRCA_positive | Delivery Date |
| Propofol | Beta_thal_or_trait | Delivery Time |
| Ripening | Blood_abnormality | GAEPIC |
| SNRI | DM1 | Delivery Method |
| SSRI | DM2 | Forceps |
| Short-acting beta agonist | DVT_or_PE | Vacuum Attempted |
| Sotalol | Diabetes | Vacuum Type |
| Suboxone | GERD | Vacuum Location |
| Succ | HIV | BreechEPIC |
| Sulfasalazine | HS | Birth Length In |
| Tacrolimus | Hemochromatosis | Birthweightm |
| Terbutaline | Hyperthyroidism | Birthweight Oz |
| Thyroid replacement | Hypothyroidism | Apgar 1 |
| Trazodone | IBD | Apgar 5 |
| Ursodiol | Migraine | Apgar 10 |
| Vancomycin | PMH_Abnormal_AFI | Delivering Clinician |
| Wellbutrin | PMH_Abnormal_genetic_finding | Pos. (L-R) |
| BLOOD PRESSURE | PMH_Abruption | Pos. (Ref.) |
| HEIGHT | PMH_Accessory_lobe | Pos. (A-P) |
| NYU R OB HEMORRAGE RISK FACTORS | PMH_Anemia | Placenta Date/Time |
| NYU R OB HEMORRAGE RISK SCALE | PMH_CHTN | Placenta Rem. |
| PULSE OXIMETRY | PMH_Cerclage | Placental App. |
| R ANE HEART RATE SPO2 | PMH_Cervical_shortening_or_insufficiency | Vessels |
| R ANE RESP RATE | PMH_Chorioangioma | Anesthesia |
| R ANE SPO2 | PMH_Circumvallate | Adtl. Del. Compl. |
| R ANE TEMPERATURE | PMH_Fibroids | Delivery Personnel |
| R BMI | PMH_GDM | Delivery Roles |
| R ESTIMATED BLOOD LOSS | PMH_GHTN | Race |
| R OB CONTRACTION DURATION | PMH_Gestational_thrombocytopenia | Abortions |
| R OB CONTRACTION FREQUENCY | PMH_Hypertensive_disorders | Augmentation Start Date |
| R OB CONTRACTION QUALITY | PMH_ICP | Augmentation Start Time |
| R OB DILATION | PMH_ITP | Baby's EDD |
| R OB EFFACEMENT | PMH_IUFD | Birth Len |
| R OB FHR BASELINE RATE | PMH_IUGR | Birth Weight |
| R OB FHR BASELINE RATE FETUS B | PMH_Invasive_placenta | Birth Wt (oz.) |
| R OB FHR BASELINE RATE FETUS C | PMH_Low_lying | ABO |
| R OB FHR CATEGORY | PMH_Macrosomia_or_LGA | Rh |
| R OB FHR CATEGORY FETUS B | PMH_Oligo | Cord Compl. |
| R OB FHR DOPPLER/FETOSCOPE RATE | PMH_PPH | C-Section Indications |
| R OB FHR PATTERN | PMH_PPROM | C/S Prior to Labor/Rupture |
| R OB FHR PATTERN FETUS B | PMH_PTL | C-Section Type |
| R OB FHR PATTERN FETUS C | PMH_Placental_abnormality | Delivery Location |
| R OB FHR VARIABILITY | PMH_Poly | Ectopic |
| R OB FHR VARIABILITY FETUS B | PMH_Pre-eclampsia_or_eclampsia | Was Fetal Demise? |
| R OB FHR VARIABILITY FETUS C | PMH_Previa | Fluid Odor |
| R OB MULTIPLE BIRTH | PMH_Retained_placenta | Forceps Attempted |
| R OB PATTERN OBSERVATIONS | PMH_SCH | Forceps Failed? |
| R OB PRESENTATION | PMH_Shoulder_dystocia | Forceps Used? |
| R OB RESTING TONE PALPATED | PMH_TTTS | Gravida |
| R OB STATION | PMH_Thrombocytopenia | Indications for Augmentation |
| RESPIRATIONS | PMH_Twin_gestation | Indications for Induction |
| TEMPERATURE | PMH_Vasa_previa | Indications for Forceps |
| WEIGHT/SCALE | Prolactinoma_or_elevated_PRL | Indications for Vacuum |
| TRANSFUSE_CRYOPRECIPITATE | Psychiatric_diagnosis | Induction Date |
| TRANSFUSE_CRYOPRECIPITATE_HEMORRHAGE | Pulmonary_HTN | Labor Induced? |
| TRANSFUSE_EMERGENT_RED_BLOOD_CELLS | Rheumatologic_disorder | Induction Time |
| TRANSFUSE_FRESH_FROZEN_PLASMA | Seizure_disorder | Rupture to Delivery |
| TRANSFUSE_FRESH_FROZEN_PLASMA_HEMORRHAGE | Short-interval_pregnancy | Antibiotics During Labor |
| TRANSFUSE_PLATELET_PHERESIS | Sickle_cell_or_trait | GA.1 |
| TRANSFUSE_PLATELET_PHERESIS_HEMORRHAGE | Stroke | Baby Sex |
| TRANSFUSE_RED_BLOOD_CELLS | TB | Delivery Date/Time |
| TRANSFUSE_RED_BLOOD_CELLS_HEMORRHAGE | Uterine_malformation | VBAC |
| RESULT_DATE_LABS_ADMISSION | Uterine_rupture_x | Live Births |
| ABO AND RH | VWF | Hx |
| ANTIBODY SCREEN | Zika_exposure | Living at Delivery Ct |
| BASOPHILS % | PRIOR_ABDOMINAL_PROCEDURE | Multiples |
| BASOPHILS ABSOLUTE | PRIOR_BARIATRIC_SURGERY | Shoulder Dystocia Occurred? |
| EOSINOPHILS % | PRIOR_CERVICAL_DILATION | Vacuum Failed? |
| EOSINOPHILS, ABSOLUTE | PRIOR_CERVICAL_PROCEDURE | Viable Birth Count |
| GRANULOCYTES IMMATURE , ABSOLUTE | HAD_PRIOR_CESAREAN_HYSTEROTOMY | Viable Birth Order |
| GRANULOCYTES, IMMATURE % | NUM_PRIOR_CESAREAN_HYSTEROTOMY | Obstetrician |
| HEMATOCRIT | ECV_WITHIN_ONE_MONTH | Para |
| HEMOGLOBIN | FETAL_PROCEDURE_PRIOR_TO_THIS_PREGNANCY | Pt Age |
| LYMPHOCYTES % | FETAL_PROCEDURES_THIS_PREGNANCY | Placenta Shared With |
| LYMPHOCYTES ABSOLUTE | PRIOR_NON_CESAREAN_UTERINE_SURGERY | PP EBL |
| MEAN CORPUSCULAR HEMOGLOBIN | MEDICAL_HISTORY_CONDITION | Preg Episode |
| MEAN CORPUSCULAR HEMOGLOBIN CONC | MEDICAL_HISTORY_ICD9 | Delivery Clinician |
| MEAN CORPUSCULAR VOLUME | MEDICAL_HISTORY_ICD10 | Delivery Date.1 |
| MEAN PLATELET VOLUME | AMA_y | Delivery Department |
| MONOCYTES % | Abruption | Delivery Rev Loc |
| MONOCYTES ABSOLUTE | Anemia | Presentation |
| NEUTROPHILS ABSOLUTE | Anticoagulation | Preterm |
| NEUTROPHILS % | Atony | Prior C-S Ct |
| NUCLEATED RBC,ABSOLUTE | BreechCLARITY | SAB |
| NUCLEATED RED BLOOD CELLS % | C-hyst | TAB |
| PLATELET COUNT | CHTN | Term |
| RDW-CV | Cerclage | Stage 3 Length |
| RDW-SD | Cervical_insufficiency | Stage 2 Length |
| RED BLOOD CELL COUNT | Cervical_shortening | Stage 1 Length |
| RED BLOOD CELL COUNT, CSF | Cholestasis | Total Labor Length |
| RED BLOOD CELL COUNT, FLUID | Chorio | TWG |
| WHITE BLOOD CELL COUNT | Chorioangioma | Triage Notes |
| Hemabate | Circumvallate | Vacuum Used? |
| Methergine | Coagulation_defect | |
