# Supplementary material for: Prediction of Maternal Hemorrhage Using Machine Learning: Retrospective Cohort Study
Source: J Med Internet Res. 2022 Jul 18;24(7):e34108. doi: 10.2196/34108 (PMC9345059; doi:10.2196/34108)
Supplement: Multimedia Appendix 2 [file jmir_v24i7e34108_app2.docx]

Supplementary material 2: 212 variables abstracted for initial model (*** = top 5 importance)

| GA_IN_DAYS | NUCLEATED_RBCa | Cervical_shortening |
| --- | --- | --- |
| Ampicillin | NUCLEATED_RBCp | Cholestasis |
| Anticoagulant | PLATELETS*** | Chorio |
| Antiepileptic | RDWCV | Coagulation_defect |
| Antibiotic | RDWSD | Excess_weight_gain |
| Antiemetic | RBC | Failed_operative_VD |
| Antihypertensive | WBC | Fibroids |
| Antiviral | GBS | GBS_diag |
| Benzodiazepine | HEMATOCRIT_FIRST_TRIMESTER | GDM |
| Betamethasone | HEMOGLOBIN_FIRST_TRIMESTER | GHTN |
| Cefazolin | HEMATOCRIT_SECOND_TRIMESTER | Grand_multip |
| Clindamycin | HEMOGLOBIN_SECOND_TRIMESTER | Ho_PPH |
| Colace | HEMATOCRIT_THIRD_TRIMESTER | Hemorrhage |
| Ephedrine | HEMOGLOBIN_THIRD_TRIMESTER | Hypertension |
| Epidural_opioid | MARITAL_STATUS | IUFD_or_demise |
| Erythromycin | RACE | IUGR |
| Gentamycin | ETHNICITY | LGA |
| H2blocker | SMOKING_TOB | Lowlying |
| Heparin | ALCOHOL_USE | Macrosomia |
| Hydralazine | ILL_DRUG_USER | Malpresentation |
| Hydroxychloroquine | AMA_x | Oligo |
| IV_opioid | APLS_or_antibodies | Operative_VD |
| Insulin | Abnormal_cervical_findings | Other_multiple |
| Insulin_aspart | All_thal | PPH |
| Insulin_regular | Asthma | PPROM |
| Labetalol | BRCA_positive | PROM |
| Magnesium | Beta_thal_or_trait | PTL |
| Nifedipine | Blood_abnormality | Poly |
| Oral_glycemic | DM1 | Preeclampsia_or_  eclampsia |
| Oral_opioid | DVT_or_PE | Previa |
| Oxytocin | Diabetes | Retained_placenta |
| PCN | GERD | SGA |
| Phenylephrine | Hyperthyroidism | Shoulder_dystocia |
| Ripening | Hypothyroidism | Succenturiate |
| SSRI | IBD | Third_degree |
| Terbutaline | Migraine | Thrombocytopenia |
| Thyroid_replacement | PMH_Anemia | Twins |
| Vancomycin | PMH_CHTN | Vanishing_Twin_or_  IUFD_or_reduction |
| BLOOD_PRESSURE_SYS | PMH_Cervical_shortening_or_insufficiency | Vasa |
| BLOOD_PRESSURE_DIAS | PMH_Fibroids | Zika |
| HEIGHT | PMH_GDM | Patient_Age |
| WEIGHT_LB | PMH_GHTN | Induction |
| BMI*** | PMH_Gestational_thrombocytopenia | Augmentation |
| PULSE_OXIMETRY | PMH_Hypertensive_disorders | Crv_Rp_Typ |
| DILATION | PMH_ICP | Rupture_Type |
| EFFACEMENT | PMH_ITP | Fluid_Color |
| FHR_BASELINE_RATE | PMH_Low_lying | Delivery_Method |
| MULTIPLE_BIRTH | PMH_Placental_abnormality | Forceps |
| PRESENTATION | PMH_Preeclampsia_or_eclampsia | Vacuum_Attempted |
| STATION | PMH_Previa | Abortions |
| RESPIRATIONS | PMH_SCH | ABO |
| TEMPERATURE2 | PMH_Thrombocytopenia | Rh |
| ANTIBODY_SCREEN | Prolactinoma_or_elevated_PRL | CS_prior_labor_rupture*** |
| BASOPHILSp | Psychiatric_diagnosis | CS_sched*** |
| BASOPHILSa | Rheumatologic_disorder | Ectopic |
| EOSINOPHILSp | Seizure_disorder | Fetal_Demise |
| EOSINOPHILSa | Sickle_cell_or_trait | Gravida |
| GRANULOCYTES_IMMATUREa | Uterine_malformation | Labor_induced |
| GRANULOCYTES_IMMATUREp | PRIOR_ABDOMINAL_PROCEDURE | Antibiotics_During_  Labor |
| HEMATOCRIT*** | PRIOR_BARIATRIC_SURGERY | Baby_Sex |
| HEMOGLOBIN | PRIOR_CERVICAL_DILATION | VBAC |
| LYMPHOCYTESp | PRIOR_CERVICAL_PROCEDURE | Live_Births |
| LYMPHOCYTESa | HAD_PRIOR_CESAREAN_HYSTEROTOMY | Living_at_del_ct |
| MCH | NUM_PRIOR_CESAREAN_HYSTEROTOMY | Multiples |
| MCHC | PRIOR_NON_CESAREAN_UTERINE_  SURGERY | Para |
| MCV | AMA_y | Preterm |
| MPV | Abruption | Prior_CS_ct |
| MONOCYTESp | Anemia | SAB |
| MONOCYTESa | Breech_diag | TAB |
| NEUTROPHILSa | CHTN | Term |
| NEUTROPHILSp | Cervical_insufficiency |  |
