# Supplementary material for: Prediction of Maternal Hemorrhage Using Machine Learning: Retrospective Cohort Study
Source: J Med Internet Res. 2022 Jul 18;24(7):e34108. doi: 10.2196/34108 (PMC9345059; doi:10.2196/34108)
Supplement: Multimedia Appendix 3 [file jmir_v24i7e34108_app3.docx]

Supplementary material 3: 76 variables abstracted for cesarean delivery model (*** = top 5 importance)

| GA_IN_DAYS*** | LYMPHOCYTESa | ALCOHOL_USE |
| --- | --- | --- |
| Antihypertensive | MCH | ILL_DRUG_USER |
| Epidural_opioid | MCHC | Abnormal_cervical_findings |
| Oxytocin | MCV | Asthma |
| Ripening | MPV | Diabetes |
| BLOOD_PRESSURE_SYS | MONOCYTESp | Psychiatric_diagnosis |
| BLOOD_PRESSURE_DIAS*** | MONOCYTESa | PRIOR_ABDOMINAL_  PROCEDURE |
| BMI*** | NEUTROPHILSa | PRIOR_CERVICAL_DILATION |
| PULSE_OXIMETRY | NEUTROPHILSp | HAD_PRIOR_CESAREAN_  HYSTEROTOMY |
| DILATION | PLATELETS*** | NUM_PRIOR_CESAREAN_  HYSTEROTOMY |
| EFFACEMENT | RDWCV | PRIOR_NON_CESAREAN_  UTERINE_SURGERY |
| FHR_BASELINE_RATE | RDWSD | Patient_Age |
| PRESENTATION | RBC | Rupture_Type |
| STATION | WBC | Fluid_Color |
| RESPIRATIONS | GBS | Abortions |
| TEMPERATURE2 | HEMATOCRIT_FIRST_TRIMESTER | ABO |
| ANTIBODY_SCREEN | HEMOGLOBIN_FIRST_TRIMESTER | CS_prior_labor_rupture |
| BASOPHILSp | HEMATOCRIT_SECOND_TRIMESTER | CS_sched |
| BASOPHILSa | HEMOGLOBIN_SECOND_TRIMESTER | Gravida |
| EOSINOPHILSp | HEMATOCRIT_THIRD_TRIMESTER*** | Labor_induced |
| EOSINOPHILSa | HEMOGLOBIN_THIRD_TRIMESTER | Live_Births |
| GRANULOCYTES_IMMATUREa | MARITAL_STATUS | Para |
| GRANULOCYTES_IMMATUREp | RACE | Prior_CS_ct |
| HEMATOCRIT | ETHNICITY | SAB |
| HEMOGLOBIN | SMOKING_TOB | Term |
| LYMPHOCYTESp |  |  |
