# Supplementary material for: Prediction of Maternal Hemorrhage Using Machine Learning: Retrospective Cohort Study
Source: J Med Internet Res. 2022 Jul 18;24(7):e34108. doi: 10.2196/34108 (PMC9345059; doi:10.2196/34108)
Supplement: Multimedia Appendix 4 [file jmir_v24i7e34108_app4.docx]

Supplementary material 4: 7 variables abstracted for 1 hour vaginal delivery model (*** = top 5 importance)

| BMI*** |
| --- |
| HEMATOCRIT*** |
| HEMOGLOBIN*** |
| PLATELETS |
| RBC |
| HEMATOCRIT_THIRD_TRIMESTER*** |
| HEMOGLOBIN_THIRD_TRIMESTER*** |
