# Supplementary material for: Prediction of Maternal Hemorrhage Using Machine Learning: Retrospective Cohort Study
Source: J Med Internet Res. 2022 Jul 18;24(7):e34108. doi: 10.2196/34108 (PMC9345059; doi:10.2196/34108)
Supplement: Multimedia Appendix 5 [file jmir_v24i7e34108_app5.docx]

Supplementary material 5: 92 variables abstracted for 2^nd^ stage vaginal delivery model (*** = top 5 importance)

| GA_IN_DAYS | MCHC | PMH_Anemia |
| --- | --- | --- |
| Antibiotic | MCV | PMH_Fibroids |
| Antiemetic | MPV | PMH_Gestational_thrombocytopenia |
| Epidural_opioid | MONOCYTESp | PMH_Placental_abnormality |
| Gentamycin | MONOCYTESa | PRIOR_ABDOMINAL_  PROCEDURE |
| Oxytocin | NEUTROPHILSa | PRIOR_CERVICAL_DILATION |
| PCN | NEUTROPHILSp | PRIOR_CERVICAL_  PROCEDURE |
| Ripening | NUCLEATED_RBCa | HAD_PRIOR_CESAREAN_  HYSTEROTOMY |
| BLOOD_PRESSURE_SYS | NUCLEATED_RBCp | Patient_Age |
| BLOOD_PRESSURE_DIAS | PLATELETS | Induction |
| BMI | RDWCV | Augmentation |
| PULSE_OXIMETRY | RDWSD | Crv_Rp_Typ |
| DILATION | RBC*** | Rupture_Type |
| EFFACEMENT | WBC | Fluid_Color |
| FHR_BASELINE_RATE | GBS | Delivery_Method |
| PRESENTATION | HEMATOCRIT_FIRST_TRIMESTER | Forceps |
| STATION | HEMOGLOBIN_FIRST_TRIMESTER | Abortions |
| RESPIRATIONS | HEMATOCRIT_SECOND_TRIMESTER | ABO |
| TEMPERATURE2 | HEMOGLOBIN_SECOND_TRIMESTER | Rh |
| ANTIBODY_SCREEN | HEMATOCRIT_THIRD_TRIMESTER*** | Fetal_Demise |
| BASOPHILSp | HEMOGLOBIN_THIRD_TRIMESTER*** | Gravida |
| BASOPHILSa | MARITAL_STATUS | Labor_induced |
| EOSINOPHILSp | RACE | Antibiotics_During_Labor |
| EOSINOPHILSa | ETHNICITY | Live_Births |
| GRANULOCYTES_IMMATUREa | SMOKING_TOB | Living_at_del_ct |
| GRANULOCYTES_IMMATUREp | ALCOHOL_USE | Para |
| HEMATOCRIT*** | ILL_DRUG_USER | Preterm |
| HEMOGLOBIN*** | Abnormal_cervical_findings | SAB |
| LYMPHOCYTESp | Asthma | TAB |
| LYMPHOCYTESa | Hypothyroidism | Term |
| MCH | Migraine |  |
